# Effect of Milking Vacuum and the Supplementation of Vitamin E and Se in Milk Quantity, Quality, and Hygiene of Mammary gland in Mountainous Greek Sheep

**DOI:** 10.3390/ani13213400

**Published:** 2023-11-02

**Authors:** Konstantinos Mamatsios, Maria-Anastasia Karatzia, Georgios Manessis, Eleni Kasapidou, Ioannis Bossis, Zoitsa Basdagianni

**Affiliations:** 1 Laboratory of Animal Husbandry, Department of Animal Production, School of Agriculture, Aristotle University of Thessaloniki, 54124 Thessaloniki, Greece; kostasmamatsios@yahoo.gr (K.M.); bossisi@agro.auth.gr (I.B.); 2 Research Institute of Animal Science, Hellenic Agricultural Organisation (HAO)-Demeter, 58100 Paralimni-Giannitsa, Greece; karatzia@elgo.gr; 3 Laboratory of Anatomy and Physiology of Farm Animals, Department of Animal Science, Agricultural University of Athens (AUA), Iera Odos 75 Str., 11855 Athens, Greece; gmanesis@aua.gr; 4 Department of Agriculture, University of Western Macedonia, 53100 Florina, Greece; ekasapidou@uowm.gr

**Keywords:** vitamin E, Se, SCC, milking ewes, milking machine vacuum

## Abstract

**Simple Summary:**

This study aimed to investigate the impact of two different machine milking vacuum levels on the quantitative and qualitative characteristics of milk and the hygiene of mammary glands in ewes, when administered vitamin E and selenium (Se). The results indicated that the chemical composition of milk was not affected by the vacuum level, but the supplementation of vitamin E and Se had a positive influence on milk quality. Milk yield was notably increased with the 46 kPa vacuum level (high vacuum level). Furthermore, the supplementation of vitamin E and Se reduced the somatic cell count (SCC), which indicated better mammary gland health status at both vacuum levels. Significant differences in total bacterial count (TBC) between the vacuum levels were not observed, regardless of vitamin E and Se administration. In conclusion, the study suggests that increased vacuum level can improve milk yields and the supplementation of animal diets with vitamin E and Se has a positive impact on milk quality as well as on reducing SCC and improving the hygiene of machine milked ewes’ mammary glands.

**Abstract:**

The aim of this research was to study the effect of two machine milking vacuum levels on the quantitative and qualitative characteristics of milk and mammary gland hygiene of ewes, when vitamin E and Se were administrated supplementarily. The experiment was conducted at the Vlasti Research Station in the Greek province of West Macedonia. Ninety-six ewes of the Mountainous Greek sheep breed were used. Animals were separated in four equal groups of 24 ewes per group. A 2 × 2 factorial design was applied, with two milking vacuum levels (38 kPa and 46 kPa) and two rations, one supplemented with vitamin E (300 I.U.) and Se (3 mg/kg DM feed) and one without any vitamin E and Se supplementation. Six test days were assigned (evening and morning milkings) at 14-day intervals, from April to July. Following milk yield control, milk samples were collected for chemical composition and somatic cell count (SCC) determination. At the end of milking of each lot, the milk from the terminal receiver of the milking machine was received for the evaluation of total bacterial count (TBC). The results revealed that milk yield was improved considerably in the case of 46 kPa vacuum level. Moreover, the chemical composition of milk was not influenced by vacuum level; however, the administration of vitamin E and Se appeared to have a positive effect. Moreover, the addition of vitamin E and Se decreased somatic cell counts (number and log_10_) at the two assessed machine milking vacuum levels. In reference to TBC and their log_10_, significant differences were not observed at both milking vacuum levels, regardless of vitamin E and Se administration. Statistical analysis did not indicate any interactions between the factors that were studied. Therefore, it is concluded that the quantity of vitamin E and Se supplemented to the ration has a positive effect on decreasing SCC and consequent positive action in the hygiene of the mammary glands of machine milked ewes.

## 1. Introduction

Milk yield and quality are crucial factors in the dairy industry, significantly impacting the overall profitability and sustainability of dairy farms. Understanding the elements that influence milk composition and yield is essential for optimizing dairy management practices. Mastitis, an inflammation of the mammary gland, holds particular importance in the context of dairy sheep farming. Moreover, recognized as the leading cause of welfare concerns in ewes [1], mastitis results in a plethora of financial issues for farmers. These include direct consequences, such as reduced milk production and indirect consequences, which encompass increased production costs due to treatment requirements and the loss of animals removed from breeding. Furthermore, mastitis affects milk quality in terms of fat, protein, and somatic cell count [2]. Proper milking practices are of paramount importance for maintaining udder health and preventing mastitis [3,4]. Vacuum level stands out as one of the most critical features of machine milking, significantly impacting udder health. It should be considered as a potential risk factor for mastitis [3,5,6,7]. Setting the vacuum level correctly ensures proper milk flow, preventing teat-end damage and clusters from falling off [8]. The vacuum level is regulated according to the milking system, whether high line or low line. Studies by [9,10,11,12] have all demonstrated that somatic cell counts tend to decrease when the vacuum level is reduced from 50 to approximately 36 to 40 kPa, and the incidence of intramammary infections is diminished [13,14]. Additionally, the proper functioning of the milking machine is essential because it can lead to cases of “over-milking” or “sub-milking” of the mammary gland, and even result in painful and stressful milking [15]. The same applies to the thorough cleaning and appropriate maintenance of the milking machine due to the potential transfer of germs through the machine’s piping.

For Greek mountainous breeds and other dairy ewes with inherently lower milk yields, achieving optimal milking efficiency involves prioritizing certain factors. One essential factor is maximizing the machine milking fraction and milk flow rate while minimizing the stripped milk fraction [12].

Utilizing a high milking vacuum can be a beneficial strategy in terms of labor intensity. When a vacuum pressure of 42 kPa is applied, a significant portion of the milk was observed to be extracted within the initial 3 min of milking, potentially reducing the overall milking time. However, it is crucial to carefully adjust and optimize the machine milking settings to prevent overmilking, as this could negate the time-saving benefits and have negative effects on the ewes’ udder health [16].

In addition to milking practices and machine settings, nutrition plays a pivotal role in dairy sheep health and milk production. Balanced nutrition with high-quality feedstuffs helps to enhance ewes’ health status and support effective mammary gland functioning. It is well known that vitamin E and selenium affect the activity of leukocytes [17,18,19] due to the micronutrients’ involvement in biological roles. Vitamin E and Se act as antioxidants and protect lipid membranes from reactive oxygen species and lipid hydroperoxides, thus improving the immune system responses [20,21]. Sheep frequently lack these two nutrients [22,23] and deficiencies of either vitamin E or Se have been associated with increased incidence and severity of intramammary infections, increases in clinical mastitis cases, and higher SCC [21,24,25]. The majority of the existing studies examine the antioxidant effects of vitamin E on sheep health and product quality, either when given alone or in combination with selenium. However, to the best of our knowledge, no research has been conducted to assess the impact of supplementing both vitamin E and Se while modifying the milking vacuum level on milk yield, milk quality, and udder health.

In light of this, the present study aims to investigate the impact of feed supplementation, specifically the administration of vitamin E (300 I.U.) and Se (3 mg/kg DM feed), in dairy ewes milked with two assigned levels of milking machine vacuum levels (38 kPa and 46 kPa), on milk yield, milk quality characteristics, and mammary gland health.

## 2. Materials and Methods

The experiment was conducted at the Vlasti Research Station in the Greek province of West Macedonia. Ninety-six dairy ewes of the mountainous Greek sheep breed (Boutsiko) were monitored during the period April-July. Animals were in their 3rd, 4th or 5th lactations (4, 5, and 6 years of age) and were divided into 4 groups of 24 animals each. The groups formed were balanced in terms of mean ewe lactation period. All parturitions had taken place within the previous December-January period and weaning had commenced 45 ± 5 days after parturition.

### 2.1. Experimental Design

The animals were divided into 4 groups, with equal mean daily milk yield and average number of lactations and remained in the assigned lots throughout the experimental period. A 2 × 2 factorial design was produced, with two levels of milking vacuum (38 kPa and 46 kPa) and two rations, basal ration (R) and the same ration with the supplementation of vitamin E (300 I.U.) and Se (3 mg/kg DM feed) (R_Vit + Se).

The basal ration consisted of 600 kg of corn, 376 kg of barley, 19 kg vitamin and mineral premix, and 4.1 kg of salt. The R_Vit + Se ration was formed by supplementing the basal ration with 600 gr vitamin E (preparation ROVIMIX E-50 Adsorbate) and 300 gr Se (formulation MicrogranTM Se 1% BMP) per ton of feed. All animals were fed 0.3 kg of wheat straw and 1.1 kg of alfalfa hay per ewe daily along with either 1.3 kg of basal ration (control groups) or 1.3 of supplemented ration (experimental groups). Animals were fed twice per day after morning and evening milkings.

Prior to the beginning of the experimentation, ewes were milked for one week with milking vacuum of 38 kPa, pulsation rate of 120 cycles/min, and pulsation ratios of 50/50 or 1/1. The experiment lasted for 75 days. In order to assess milk yields and composition, samples from both afternoon and morning milkings were collected every 14 days. Two milkings for each sampling occasion were performed within 24 h, the first at 19:30 in the afternoon and the second at 7:30 in the morning of the following day. A constant interval of twelve hours between milkings was kept throughout the study.

Milking began by placing the cups without any prior special handling of the mammary gland and continued by hand stripping for about 10 s, with the cups remaining attached to the teats. At the end of milking, milk yield was recorded for each ewe and an individual milk sample was collected for chemical composition analysis and for SCC determination.

At the end of milking for each group, bulk milk samples were collected from the terminal receiver of the milking machine in special sterile tubes for total TBC evaluation. All these samples were frozen immediately at −18 °C to be conserved until the analysis.

### 2.2. Machine Milking

The milking parlor was of the modern “Casse” system type (fast exit) with 2 × 24 places and 2 × 12 milking units. Milking machine working parameters were characterized by a high line of milk and vacuum, internal silicone milking cups, plastic cups (barrel), vacuum levels of 38 kPa and 46 kPa, speed (frequency) pulses of 120/min, and suction ratio of 1:1 or 50:50.

### 2.3. Milk Recording and Analysis of Milk Composition and Somatic Cell Count

The following milk fractions from the afternoon and morning milkings were recorded every 14 days:

- Machine Milk (MM): representing the quantity of milk obtained after milking cup attachment, until milk flow stopped;
- Stripping Milk (SM): representing the quantity of milk obtained after mammary stripping by hand, while milking cups are still attached;
- Total Milk (TM): representing the whole quantity milked.

The MM and TM fractions were calculated during milking. Stripping milk was estimated using the operation: SM = TM − MM.

Evening and morning milk samples from the TM of each ewe were pooled together to determine the chemical composition and SCC. Milk composition [milk fat, protein, lactose, total solids (TS), and solid non-fat (SnF)] were analyzed using Milcoscan™ 4000 (Foss Company, Hillerød, Denmark) and somatic cell counts were estimated by Fossomatic™ 5000/FC (Foss Company).

### 2.4. Determination of Milk Total Bacterial Count (TBC)

Prior to TBC analysis, the milk samples were thawed under refrigeration (4 °C) and TBC was measured within 24 h of thawing. To control the total viable milk flora, plate count agar (PCA) (Bioprepare microbiology Company, Keratea - Attica, GR) was used by the inclusion technique and plates were incubated at 30 °C for 72 h in aerobic conditions (International Organization for Standardization (ISO 4833: 2003) [26]). The sterilized medium was collected in 100 mL vials under freezing temperatures and after boiling for liquefaction, it was partitioned equally into 5 plates under aseptic conditions, at about 20 mL/plate. Successive dilutions were performed using sterile Ringer fluid (Bioprepare microbiology Company, GR). For inoculation, dilutions of 10^−3^ and 10^−4^ were used (according to the values of TBC reported in literature and tests performed at the start of the experiment).

## 3. Statistical Analysis

Multivariate analysis of variance (MANOVA) using the general linear model procedure of SPSS software (version 26.0, SPSS Inc., Chicago, IL, USA) was applied to evaluate the effect of vacuum level on both diets and on all the parameters under study. The main effects of the two experimental factors as well as their interactions were examined. The SCC and TBC values were normalized using the log_10_ transformation.

## 4. Results

The effect of rations on milk chemical composition milk at both vacuum levels is presented in Table 1.

At the 38 kPa vacuum level, the supplementation of vitamin E and Se resulted in significantly (*p* < 0.05) higher concentrations in several milk components compared to the control group without supplementation. Specifically, the fat content increased from 6.70% to 7.38%, lactose concentration increased from 4.50% to 4.66%, total solids increased from 18.59% to 19.56%, and solid non-fat content increased from 11.61% to 11.89%. However, there were no significant differences in the protein percentage between the two diets at this vacuum level (Table 1). Fat exhibited a decrease from 6.93% to 6.73%, protein increased from 6.01% to 6.17%, and lactose from 4.54% to 4.61% between R and R + VitE_Se diets (*p* < 0.05), respectively. The total solids showed a small increase from 18.84 to 18.87% and the residual solid non-fat increased from 11.63 to 11.86% (Table 1). No significant differences were observed between the two vacuum levels except in fat content with higher concentrations at a vacuum level of 38 kPa compared to 46 kPa (7.38% vs. 6.73%) (*p* < 0.05) (Table 1). From the statistical analysis of this study, the effect of the two vacuum levels on both diets did not reveal any interactions between the examined experimental factors.

**Figure 1:**
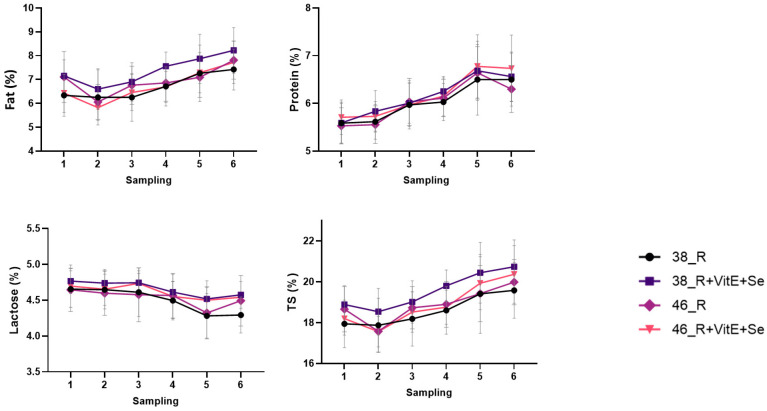
Mean (±SE) of fat, protein, lactose, and total solids content throughout the experimental period.

The effect of vitamin E plus Se supplementation and vacuum level during the six sampling occasions for the four lots of animals on fat, protein, lactose, and total solids content are presented in Figure 1.

The observed increase in fat percentage in the R + VitE_Se group, apart from the second sampling occasion, is evident in all groups with the 38 kPa group. The consistent increase in protein percentage across all experimental groups indicates a general trend of increasing protein content as lactation progressed. This aligns with typical lactation curves, which show an upward trend in milk protein content during early lactation.

On the contrary, lactose content showed a declining trend, with the lowest values in the groups without vitamin E and Se. Total solids exhibited an increase, with the highest values observed in the groups with vitamin E and Se supplementation, regardless of vacuum level.

Table 2 displays the impact of vitamin E and Se supplementation, as well as vacuum level, on milk SCC and TBC. Significant differences (*p* < 0.05) in SCC between the two diets were observed at both vacuum levels. Specifically, SCC decreased from 1,202,569 cells/mL to 438,190 cells/mL at the 38 kPa vacuum level and from 1,113,467 cells/mL to 485,616 cells/mL at the 46 kPa vacuum level for rations R and R + VitE_Se, respectively. Furthermore, the log_10_ transformation of SCC values followed the same pattern, with a statistically significant reduction (*p* < 0.05) in the values from 5.58 to 5.32 at the 38 kPa vacuum level and from 5.62 to 5.37 at the 46 kPa vacuum level for rations R and R + VitE_Se, respectively. No significant differences (*p* > 0.05) were found in TBC values between the two diets in any vacuum level.

Table 3 presents the results concerning the influence of vitamin E and selenium (Se) supplementation, as well as the two vacuum levels, on milk production. Specifically, at the vacuum level of 38 kPa, notable alterations in machine milk (MM) and total milk (TM) were observed, both of which exhibited a statistically significant increase (*p* < 0.05). Machine milk increased from an initial volume of 417.5 mL to 440.2 mL, while total milk increased from an initial volume of 563.9 mL to 579.2 mL. Regarding stripped milk (SM), a reduction in milk yield was observed when comparing the two diets at both vacuum levels. However, it is important to note that this reduction was only statistically significant at 46 kPa (*p* < 0.05). In more specific terms, when the vacuum level was set at 38 kPa, SM showed a non-statistically significant decrease from 146.4 mL to 139 mL. On the other hand, at the 46 kPa vacuum level, there was a significant reduction (*p* < 0.05) in SM yield, decreasing from 155.8 mL to 144.3 mL.

**Figure 2:**
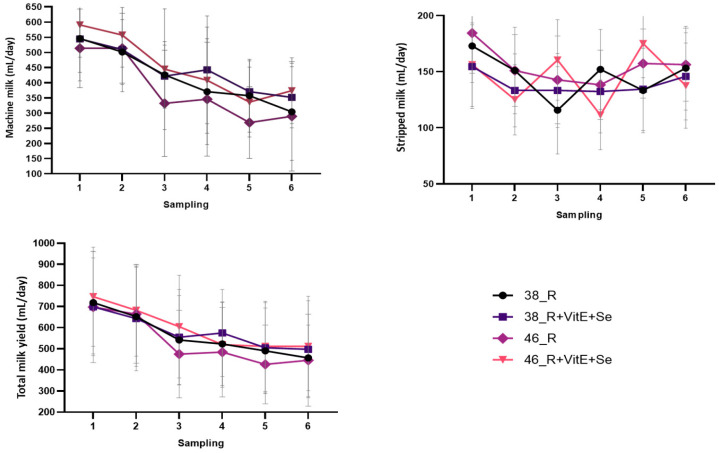
Mean (±SE) of fractional and overall milk production throughout the experimental period.

The effect of vitamin E plus Se supplementation and vacuum level during the six sampling occasions for the four lots of animals on machine milk, stripped milk, and total milk is presented in Figure 2.

The stripped milk displayed an uncertain pattern throughout the experiments, whereas the total milk and machine milk amounts decreased over the course of the study. This decrease is expected as it corresponds with the natural decline in milk production as lactation progresses.

## 5. Discussion

Our goal was to evaluate the impact of feed supplementation, specifically the administration of vitamin E (300 I.U.) and Se (3 mg/kg DM feed), on milk yield, milk quality characteristics, and mammary gland health in dairy ewes milked under two assigned vacuum levels (38 kPa and 46 kPa).

The administration of vitamin Ε and selenium (Se) in ewes modified the chemical composition of milk by increasing the concentration of some of the milk parameters under study, regardless of the vacuum level (38 and 46 kPa), in contrast to the results obtained by Pulido et al. (2012) [27] on milk composition when following dietary supplementation of α-tocopherol plus selenium in Assaf ewes. In this study, fat content and total solids were affected by diet supplementation only at the 38 kPa vacuum level group [6.70% vs. 7.38% (*p* < 0.05) and 18.59 vs. 19.56 (*p* < 0.001), respectively]. At 46 kPa, a significantly higher (*p* < 0.05) level of protein content was recorded when vitamin E and Se were administered. However, no significant differences in protein content were observed between the two diets at the 38 kPa vacuum level. Both lactose content and solid non-fat content (SnF) increased significantly (*p* < 0.05) with the supplementation of vitamin E and Se at both vacuum levels. We can assume that the observed increases in basic components in milk derived from ewes fed with the R + VitE_Se ration can be attributed to better utilization of nutrients.

The results also showed that protein, lactose, total solids, and SnF content of milk were not influenced by vacuum level, further supporting the evidence of previous studies in dairy sheep [5,28] and goats [16]. In contrast, milk fat content was significantly affected by the vacuum level in this study, which was also supported by Caria et al. (2021) in Alpine goats [29] and Mohamed et al. (2021) in the Sicilo-Sarde ewes [30]. A probable explanation is that low vacuum levels lengthen milking duration and encourage the extraction of alveolar milk, which is the richest in fat.

Concerning SCC, no significant differences were observed between the two vacuum levels, in agreement with the results of Peris et al. (2003) [13] in sheep and Zucali et al. (2019) [16] in goats. On the contrary, Sinapis et al. (2006) [12] reported an increase in SCC in Boutsiko ewes when comparing three vacuum levels of 38, 44, and 50 kPa. However, when vitamin E and Se were added to ewes’ diet, a significant decrease (*p* < 0.001) in SCC values at both vacuum levels was observed. The results indicate that the supplementation with vitamin E and Se has a positive impact on reducing SCC in milk. SCC is a widely used indicator of udder health in dairy animals, and lower SCC values generally suggest better udder health and higher milk quality [25]. Vitamin E is a powerful antioxidant that helps protect the cells from oxidative damage and plays a crucial role in maintaining the integrity of cell membranes and enhancing immune responses [31,32,33]. Selenium, on the other hand, is an essential trace mineral that acts as a cofactor for various enzymes involved in antioxidant defense and immune function [34].

A significant difference in daily milk yield (TM) and machine milk (MM) between the two vacuum levels used was not detected, which is consistent with previous findings by Lacetera et al. (1999) [35] for Sardinian ewes and Sinapis et al. (2006) [12] for the same breed studied here (Boutsiko). However, the administration of vitamin E and selenium (Se) in ewes’ diet led to a significant (*p* < 0.05) increase in MM (417.5 vs. 440.2 at 38 kPa and 377.3 vs. 451.9 at 46 kPa) and TM (563.9 vs. 579.2 at 38 kPa and 532.3 vs. 596.2 at 46 kPa). This agrees with the findings of Lacetera et al. (1999) and Tufarelli et al. (2011) [35,36] in studies with sheep and goats, respectively. Regarding stripped milk (SM), a significant difference was also observed between the two diets at 46 kPa, but this parameter was affected by the vacuum level in the R diet (146.4 mL vs. 155.8 mL). Additionally, the study revealed that with the increase in vacuum level from 38 to 46 kPa, the percentage of MM on total milk yield declined from 74% to 71%, while the percentage of SM on total milk yield increased from 26% to 29%, in accordance with the observations made by Marnet et al. (1996) [37] and Sinapis et al. (2006) [12]. Interestingly, this effect was observed only in the ewes that did not receive vitamin E and selenium supplements. However, under supplementation of vitamin E and Se, these percentages were similar (76.00% and 75.79% of MM on total milk yield and 23.99% and 24.20% of SM on total milk yield) at both vacuum levels. The different responses of the ewes regarding the groups receiving supplements (R_Vit + Se) may be explained due to the significant increase in the MM milk yield and decreased SM milk yield could be attributed to the more effective machine milking process. The results suggest that the addition of vitamin E and selenium can enhance milk production and milking efficiency with desirable effects during milking while ensuring a high percentage of MM fraction and a low percentage of SM fraction as these two factors are essential for dairy ewes with inherently lower milk yields.

## 6. Conclusions

In conclusion, the findings of this study suggest that the chemical composition of milk in dairy ewes is not influenced by the vacuum level. Moreover, the supplementation of vitamin E and selenium in ewes’ diet significantly enhanced the chemical composition of milk, including fat, total solids, protein, lactose, and solid non-fat content. This improvement in milk composition can be attributed to the increased utilization of nutrients. Furthermore, the study demonstrated that the addition of vitamin E and selenium resulted in a significant decrease in somatic cell counts (SCC), indicating improved udder health and milk quality. SCC is an important indicator of udder health in dairy animals, and lower values are generally associated with better milk quality. The decrease in SCC was quantified by comparing the values before and after vitamin E and selenium supplementation, revealing a significant reduction in somatic cells per milliliter of milk. However, there were no significant differences in total bacterial count (TBC) concentrations attributed to diet supplementation. Additionally, vacuum level did not affect daily milk yield or machine milk, but the supplementation of vitamin E and selenium significantly increased machine milk and total milk yield at a vacuum level of 46 kPa. The study found that as the vacuum level increased, machine milk percentage declined and stripping milk percentage increased, but this effect was observed only in ewes that did not receive vitamin E and Se supplements. Overall, the results highlight the potential benefits of supplementing ewes’ diets with vitamin E and selenium in improving milk composition, reducing SCC, and enhancing milk yield along with machine milking efficiency. Utilizing effectively the results of the present study, further research will include more dairy sheep breeds and the application of the suggested nutritional supplementation, towards an advanced managerial practice, interconnecting nutrition and milking for the advancement of milk quality characteristics.

## Figures and Tables

**Table 1:** The effect of vitamin E plus Se supplementation and vacuum level on the chemical composition of milk.

Items	38 kPa		46 kPa		SED_V_	SED_D_	Effects		
	R	R + VitE_Se	R	R + VitE_Se			V	D	V × D
Fat (%)	6.70 ^a^	7.38 ^b^	6.93 ^ac^	6.73 ^ad^	0.16	0.12	0.006	0.004	0.043
Protein (%)	6.03 ^a^	6.15 ^a^	6.01 ^ac^	6.17 ^ad^	0.11	0.07	0.932	0.003	0.712
Lactose (%)	4.50 ^a^	4.66 ^b^	4.54 ^ac^	4.61 ^bd^	0.07	0.04	0.896	0.002	0.157
TS (%)	18.59 ^a^	19.56 ^b^	18.84 ^ab^	18.87 ^ab^	0.13	0.17	0.077	0.000	0.048
SnF	11.61 ^a^	11.89 ^b^	11.63 ^ac^	11.86 ^bd^	0.13	0.07	0.869	0.000	0.694

R: basal ration; R + Vit_Se: the same ration with the supplementation of vitamin E (300 I.U.) and Se (3 mg/kg DM feed); SEDv: standard error of difference between the two vacuums; SED_D_ standard error of difference between the two diets; V: the *p*-value of the effect of vacuum; D: the *p*-value of the effect of diet; V × D: the *p*-value of the effect of the interaction between diet and vacuum. The different letters in the same line indicate significant differences (*p* < 0.05).

**Table 2:** The effect of vitamin E plus Se supplementation and vacuum level on SCC and TBC of milk.

Items	38 kPa		46 kPa		SED_V_	SED_D_	Effects		
	R	R + VitE_Se	R	R + VitE_Se			V	D	V × D
SCC/mL	1,202,569 ^a^	438,190 ^b^	1,113,467 ^abc^	485,616 ^abd^	229,918.63	285,560.66	0.904	0.000	0.779
log_10_ SCC	5.58 ^a^	5.32 ^b^	5.62 ^abc^	5.37 ^abd^	0.06	0.05	0.304	0.000	0.939
TBC(cfu/mL^1^)	268.50	243.15	220.00	197.00	60,012.26	64,525.05	0.604	0.316	0.908
log_10_ TBC	5.38	5.33	5.32	5.26	0.11	0.10	0.462	0.447	0.959

R: basal ration; R + Vit_Se: the same ration with the supplementation of vitamin E (300 I.U.) and Se (3 mg/kg DM feed); SEDv: standard error of difference between the two vacuums; SED_D_: standard error of difference between the two diets; V: the *p*-value of the effect of vacuum; D: the *p*-value of the effect of diet; V × D: the *p*-value of the effect of the interaction between diet and vacuum. The different letters in the same line indicate significant differences (*p* < 0.05).

**Table 3:** The effect of vitamin E plus Se supplementation and vacuum level on fractional and overall milk production.

Items	38 kPa		46 kPa		SED_V_	SED_D_	Effects		
	R	R + VitE_Se	R	R + VitE_Se			V	D	V × D
MM (mL/day)	417.5 ^a^	440.2 ^b^	377.3 ^abc^	451.9 ^abd^	25.26	24.87	0.828	0.006	0.272
SM (mL/day)	146.4 ^a^	139.0 ^ab^	155.8 ^cb^	144.3 ^ad^	7.17	7.00	0.028	0.039	0.519
TM (mL/day)	563.9 ^a^	579.2 ^b^	532.3 ^abc^	596.2 ^abd^	27.01	26.11	0.712	0.045	0.400

R: basal ration; R + Vit_Se: the same ration with the supplementation of vitamin E (300 I.U.) and Se (3 mg/kg DM feed); SEDv: standard error of difference between the two vacuums; SED_D_: standard error of difference between the two diets; V: the *p*-value of the effect of vacuum; D: the *p*-value of the effect of diet; V × D: the *p*-value of the effect of the interaction between diet and vacuum; MM: machine milk (mL/day); SM: stripped milk (mL/day); TM: total milk yield (mL/day). The different letters in the same line indicate significant differences (*p* < 0.05).

## Data Availability

The data presented in this study are available on request from the corresponding author. The data are not publicly available due to the trial being a private initiative and the funders having no role in the design of the study; in the collection, analyses, or interpretation of data; in the writing of the manuscript; or in the decision to publish the results.
